# Supplementary material for: Short, Lipidated Dendrimeric γ-AApeptides as New Antimicrobial Peptidomimetics
Source: Int J Mol Sci. 2023 Mar 29;24(7):6407. doi: 10.3390/ijms24076407 (PMC10094648; doi:10.3390/ijms24076407)
Supplement: Supplementary file 1 [file ijms-24-06407-s001.zip › ijms-2244699-SI.pdf]

Supporting information

Short, Lipidated Dendrimeric γ-AApeptides as New Antimicrobial Peptidomimetics

Yafeng Wang [1], Menglin Xue [1], Ruixuan Gao [1], Soumyadeep Chakraborty [2], Shaohui Wang [2], Xue Zhao [2], Meng Gu [2], Chuanhai Cao [3], Xingmin Sun [2] and Jianfeng Cai [1,*]

[1] Department of Chemistry, University of South Florida, 4202 E. Fowler Ave., Tampa, FL 33620, USA
[2] Department of Molecular Medicine, Morsani College of Medicine, University of South Florida, 12901 Bruce B Downs Blvd., Tampa, FL 33612, USA
[3] College of Pharmacy, University of South Florida, 12901 Bruce B Downs Blvd., Tampa, FL 33612, USA
* Correspondence: jianfengcai@usf.edu

1. GENERAL SYNTHESIS OF COMPOUND 2

2. HPLC DATA 2

3. ^{1}H NMR SPECTRUM AND ^{13}C NMR SPECTRUM 5

4. TABLE S1: HRMS DATA 14

1. General Synthesis of Compound

The Fmoc-protected amino acids were bought from Chemimpex, and the reagents used in the reaction were bought from Fisher Scientific and Sigma Aldrich. The building blocks were synthesized according to a previously published paper [1]. Next, the building block was loaded on Rink-Amide beads with DIC and HOBt in DMF, reacting for 4 hours. After deprotecting the Fmoc protecting, palmitic acid was coupled with DIC and HOBt again. The Alloc protecting group of the secondary amine was removed with dimethylamine boron ((CH3)2NH.BH3), and tetrakis (triphenylphosphine) palladium, the second building, was coupled similarly to the first building block. The Alloc protecting group was deprotected again, then the couple Fmoc-protected beta alanine was also deprotected. Subsequently, the Fmoc was removed, and the beads were cleaved with 50% TFA in DCM. The solvent was removed, and the solid was precipitated with cold ether. Finally, the oil was purified by HPLC, and the final product was obtained.

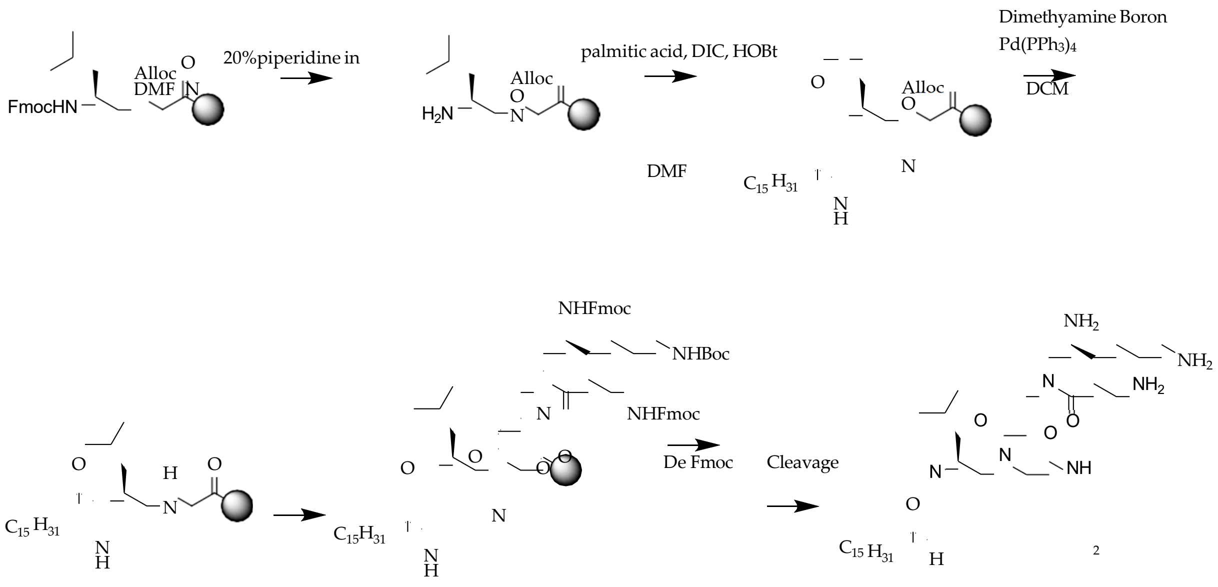

2. HPLC Data

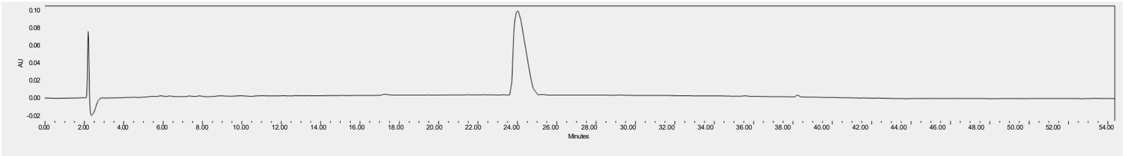

YW-1

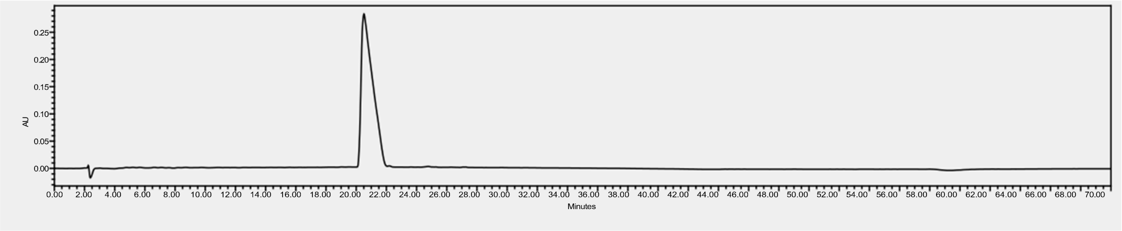

YW-2

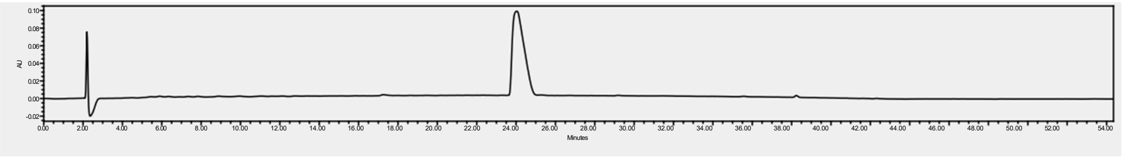

YW-3

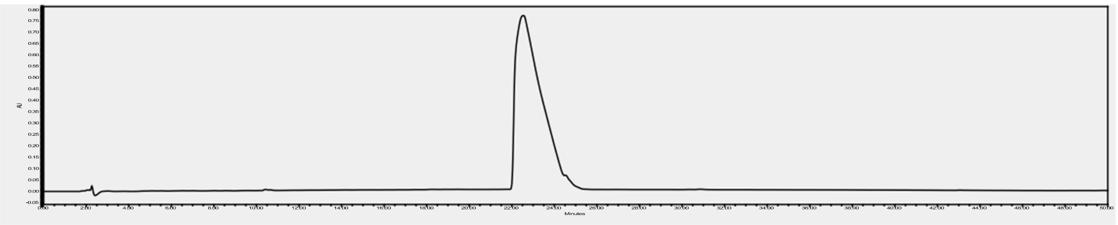

YW-4

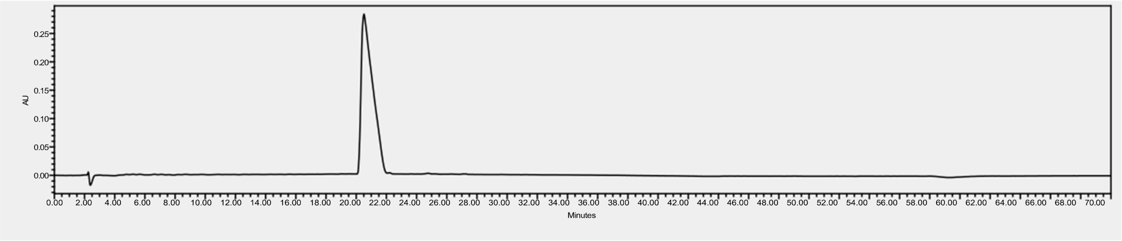

YW-5

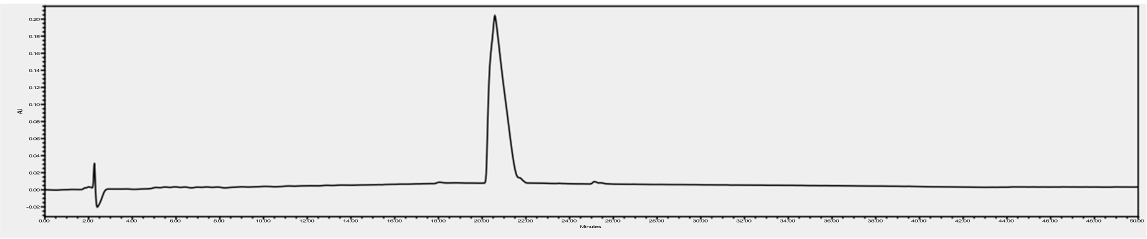

YW-6

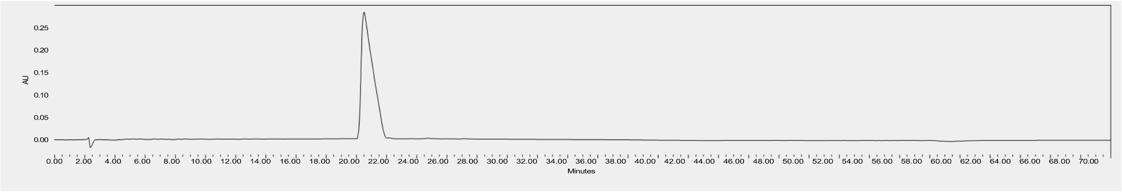

YW-7

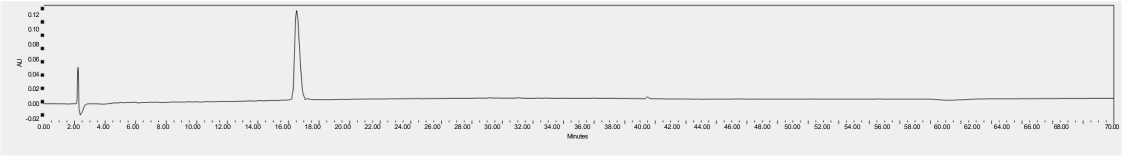

YW-8

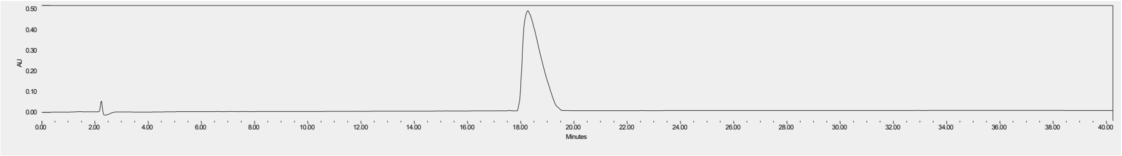

YW-9

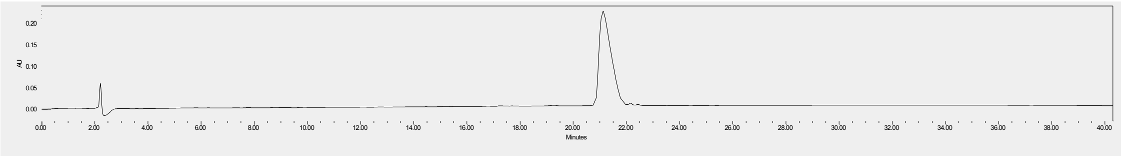

YW-10

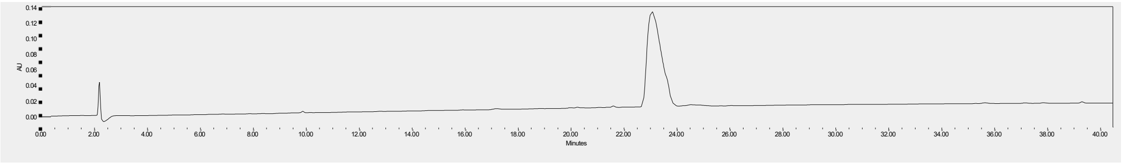

YW-11

3. 1H NMR Spectrum and 13C NMR Spectrum

YW-1:

^{1}H NMR (600 MHz, DMSO-d_6) δ 8.09 (s, 1H), 7.96 – 7.71 (m, 9H), 7.57 (d, *J* = 8.9 Hz, 1H), 7.37 (d, *J* = 22.1 Hz, 1H), 7.17 (d, *J* = 12.8 Hz, 1H), 6.99 (d, *J* = 14.9 Hz, 1H), 4.10 – 3.88 (m, 3H), 3.74 (d, *J* = 16.5 Hz, 1H), 3.50 (ddd, *J* = 33.1, 14.7, 7.5 Hz, 4H), 3.26 – 3.16 (m, 1H), 3.02 (qd, *J* = 6.4, 3.2 Hz, 2H), 2.75 (dddd, *J* = 28.1, 13.9, 6.9, 3.1 Hz, 5H), 2.11 – 1.95 (m, 2H), 1.64 – 1.30 (m, 10H), 1.24 (s, 26H), 0.89 – 0.83 (m, 6H), 0.80 (dd, *J* = 10.4, 6.5 Hz, 3H).

^{13}C NMR (151 MHz, DMSO-d_6) δ 172.72, 172.49, 170.78, 158.82, 158.61, 38.94, 36.02, 35.73, 31.74, 30.46, 29.50, 29.15, 28.97, 27.14, 24.78, 23.80, 22.55, 22.01, 21.72, 14.41.

YW-2:

^{1}H NMR (600 MHz, DMSO-d_6) δ 8.20 – 7.96 (m, 1H), 7.94 – 7.71 (m, 9H), 7.30 – 7.10 (m, 6H), 4.27 – 4.09 (m, 1H), 4.08 – 3.94 (m, 1H), 3.93 – 3.75 (m, 1H), 3.56 (ddd, *J* = 87.1, 13.0, 5.8 Hz, 4H), 3.02 (p, *J* = 6.4 Hz, 2H), 2.94 (dd, *J* = 13.4, 7.4 Hz, 1H), 2.85 – 2.53 (m, 7H), 2.45 (q, *J* = 7.0 Hz, 1H), 1.93 (td, *J* = 7.3, 4.0 Hz, 2H), 1.53 (dtt, *J* = 17.2, 10.4, 5.2 Hz, 4H), 1.45 – 1.10 (m, 27H), 1.09 – 0.94 (m, 2H), 0.85 (t, *J* = 6.9 Hz, 3H).

^{13}C NMR (151 MHz, DMSO-d_6) δ 171.27, 170.78, 158.54, 129.45, 128.50, 128.39, 118.54, 116.71,49.99, 47.31, 44.32, 38.95, 31.75, 29.52, 29.38, 29.16, 27.17, 25.67, 22.55, 22.02, 14.42.

YW-3:

^{1}H NMR (600 MHz, DMSO-d_6) δ 7.95 – 7.69 (m, 9H), 4.07 – 3.76 (m, 3H), 3.50 (dd, *J* = 17.8, 10.9 Hz, 4H), 3.24 (dd, *J* = 7.3, 3.6 Hz, 1H), 3.01 (h, *J* = 6.2 Hz, 2H), 2.85 – 2.63 (m, 5H), 2.02 (dq, *J* = 12.1, 7.0 Hz, 2H), 1.62 – 1.49 (m, *J* = 6.5 Hz, 4H), 1.49 – 1.35 (m, 4H), 1.24 (s, 24H), 1.05 (d, *J* = 6.7 Hz, 1H), 0.97 (d, *J* = 6.7 Hz, 1H), 0.85 (t, *J* = 6.9 Hz, 3H).

^{13}C NMR (151 MHz, DMSO-d_6) δ 172.19, 171.93, 158.82, 158.62, 117.72, 116.43, 49.91, 47.31, 43.58, 38.95, 35.74, 31.75, 30.39, 29.51, 29.47, 29.16, 27.16, 25.70, 22.55, 22.01, 18.70, 18.46, 14.42.

YW-4

^{1}H NMR (600 MHz, DMSO-d_6) δ 8.11 (d, *J* = 7.8 Hz, 1H), 7.98 – 7.68 (m, 9H), 7.03 – 6.91 (m, 3H), 6.67 – 6.58 (m, 2H), 4.17 – 3.75 (m, 3H), 3.57 (td, *J* = 13.7, 6.2 Hz, 2H), 3.41 – 3.28 (m, 3H), 3.25 (dd, *J* = 15.1, 9.2 Hz, 1H), 3.01 (h, *J* = 6.2 Hz, 2H), 2.92 (dd, *J* = 13.4, 7.7 Hz, 1H), 2.83 – 2.56 (m, 6H), 2.43 (dd, *J* = 13.5, 9.3 Hz, 1H), 1.95 (tt, *J* = 7.6, 3.1 Hz, 2H), 1.55 (tt, *J* = 14.0, 5.2 Hz, 4H), 1.46 – 1.30 (m, 4H), 1.30 – 1.13 (m, 22H), 1.09 (h, *J* = 7.0 Hz, 2H), 0.85 (t, *J* = 7.0 Hz, 3H).

^{13}C NMR (151 MHz, DMSO-d_6) δ 172.62, 171.79, 170.95, 158.97, 158.76, 156.14, 130.27, 128.92, 118.51, 116.53, 115.34, 115.23, 49.92, 47.40, 38.93, 37.31, 36.07, 35.72, 31.75, 29.47, 29.17, 27.16, 25.68, 22.55, 22.02, 14.41.

YW-5

^{1}H NMR (600 MHz, DMSO-d_6) δ 8.03 (s, 1H), 7.89 – 7.63 (m, 9H), 4.08 – 3.76 (m, 4H), 3.57 – 3.38 (m, 6H), 3.03 (dp, *J* = 17.3, 6.8, 6.0 Hz, 3H), 2.84 – 2.63 (m, 6H), 2.10 – 1.99 (m, 2H), 1.53 (h, *J* = 6.4, 5.4 Hz, 4H), 1.43 (dt, *J* = 30.3, 7.3 Hz, 4H), 1.24 (s, 24H), 0.86 (t, *J* = 6.9 Hz, 3H).

^{13}C NMR (151 MHz, DMSO-d_6) δ 172.75, 171.00, 159.29, 158.54, 50.10,49.42, 38.97, 35.75, 31.75, 30.40, 29.53, 29.31, 29.17, 27.18, 25.72, 22.56, 14.42.

YW-6:

^{1}H NMR (600 MHz, DMSO-d_6) δ 8.06 (s, 1H), 7.78 (d, *J* = 31.6 Hz, 13H), 3.95 (dd, *J* = 20.6, 4.1 Hz, 2H), 3.88 (d, *J* = 14.1 Hz, 1H), 3.72 (d, *J* = 16.5 Hz, 1H), 3.58 – 3.40 (m, 4H), 3.01 (s, 2H), 2.95 (dd, *J* = 13.7, 8.3 Hz, 1H), 2.76 (dtd, *J* =

31.4, 17.3, 14.7, 7.3 Hz, 8H), 2.45 – 2.35 (m, 1H), 2.03 (dq, *J* = 9.5, 7.0, 6.6 Hz, 2H), 1.62 – 1.36 (m, 13H), 1.24 (s, 31H), 0.86 (t, *J* = 6.9 Hz, 3H).

^{13}C NMR (151 MHz, DMSO-d_6) δ 171.75, 169.58, 159.06, 158.32, 47.62, 45.31, 43.94, 39.03, 31.75, 29.54, 29.17, 22.55, 14.42.

YW-1:

^{1}H NMR (600 MHz, DMSO-d_6) δ 8.05 (s, 1H), 7.95 – 7.53 (m, 11H), 4.09 – 3.84 (m, 3H), 3.73 (d, *J* = 16.5 Hz, 1H), 3.51
(s, 6H), 3.25 (s, 2H), 3.10 – 2.88 (m, 4H), 2.85 – 2.63 (m, 5H), 2.46 – 2.35 (m, 1H), 2.02 (dq, *J* = 10.1, 6.9 Hz, 2H), 1.60 – 1.05 (m, 38H), 0.85 (t, *J* = 6.9 Hz, 3H).

^{13}C NMR (151 MHz, DMSO-d_6) δ 172.04, 171.95, 158.82, 158.66, 157.24, 118.85, 117.36, 50.22, 47.53, 44.10, 39.00,
36.22, 36.07, 35.73, 31.75, 29.54, 29.17, 22.55, 14.42.

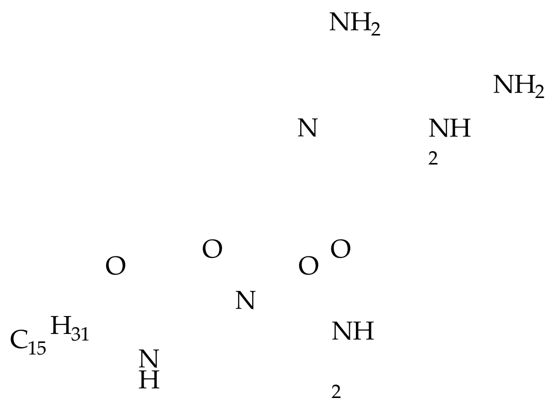

YW-1

YW-1 carbon.10.fid
C13CPD DMSO /mnt/d2/iconnmr/data/sxue sxue 21

17[illegible].01 17[illegible].52 17[illegible].78 15[illegible].82 15[illegible].61 118.33 116.39 79.16 49.90 45.82 45.73 44.46 38.94 35.73 31.74 29.50 29.15 27.14 25.76 24.78 23.80 22.55 21.72 14.41

4500
4000
3500
3000
2500
2000
1500
1000
500

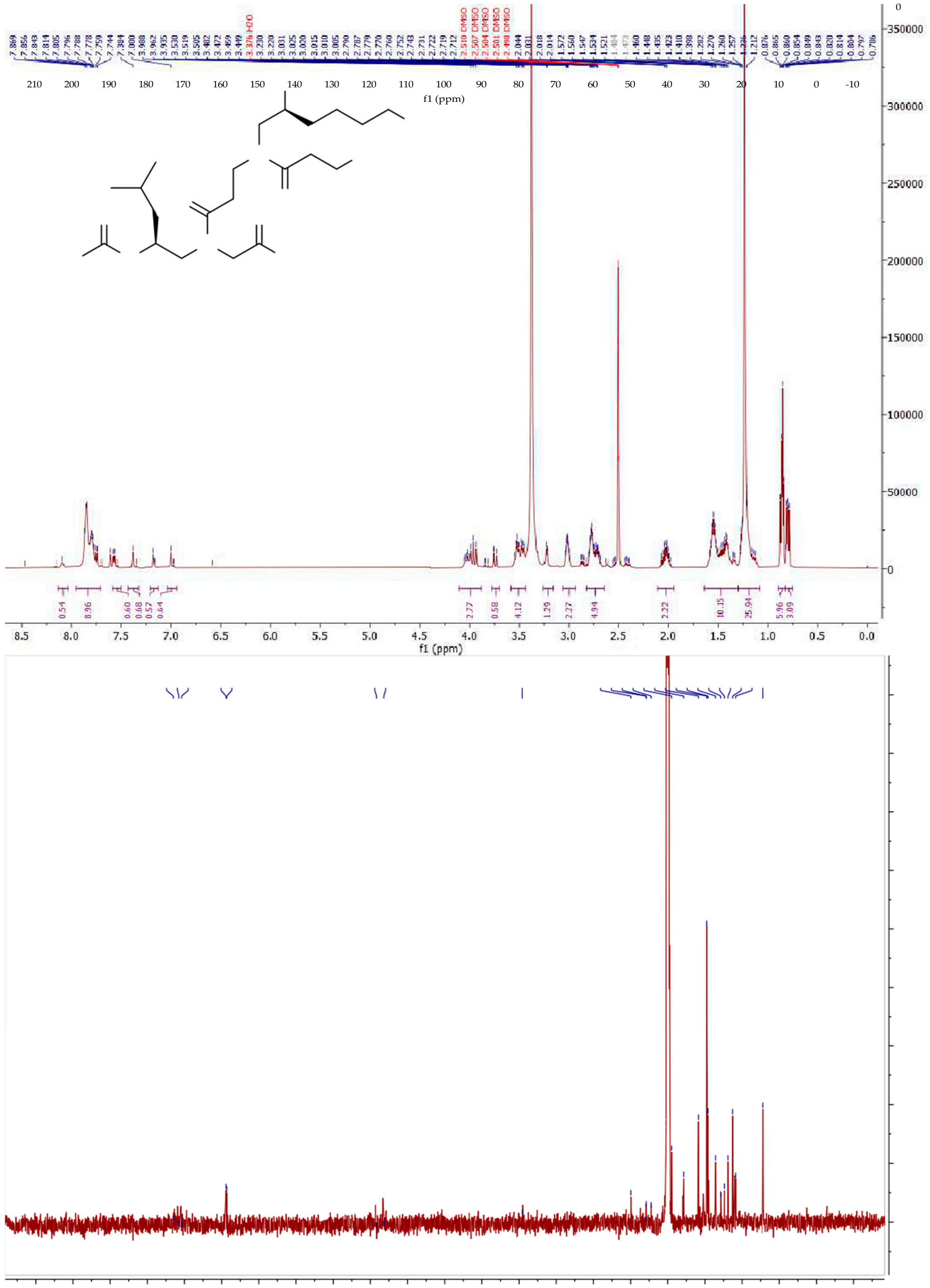

f1 (ppm)

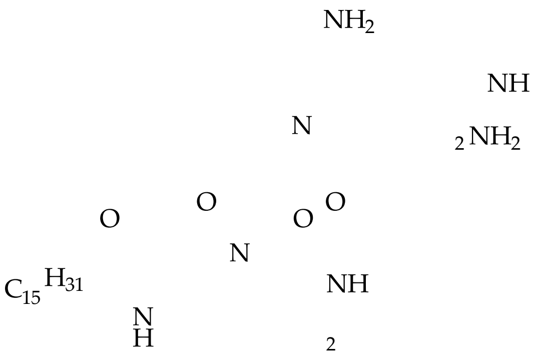
NH2
NH
N
2 NH2
O
O
O
O
N
C15H31
NH
N
H
2

YW-2

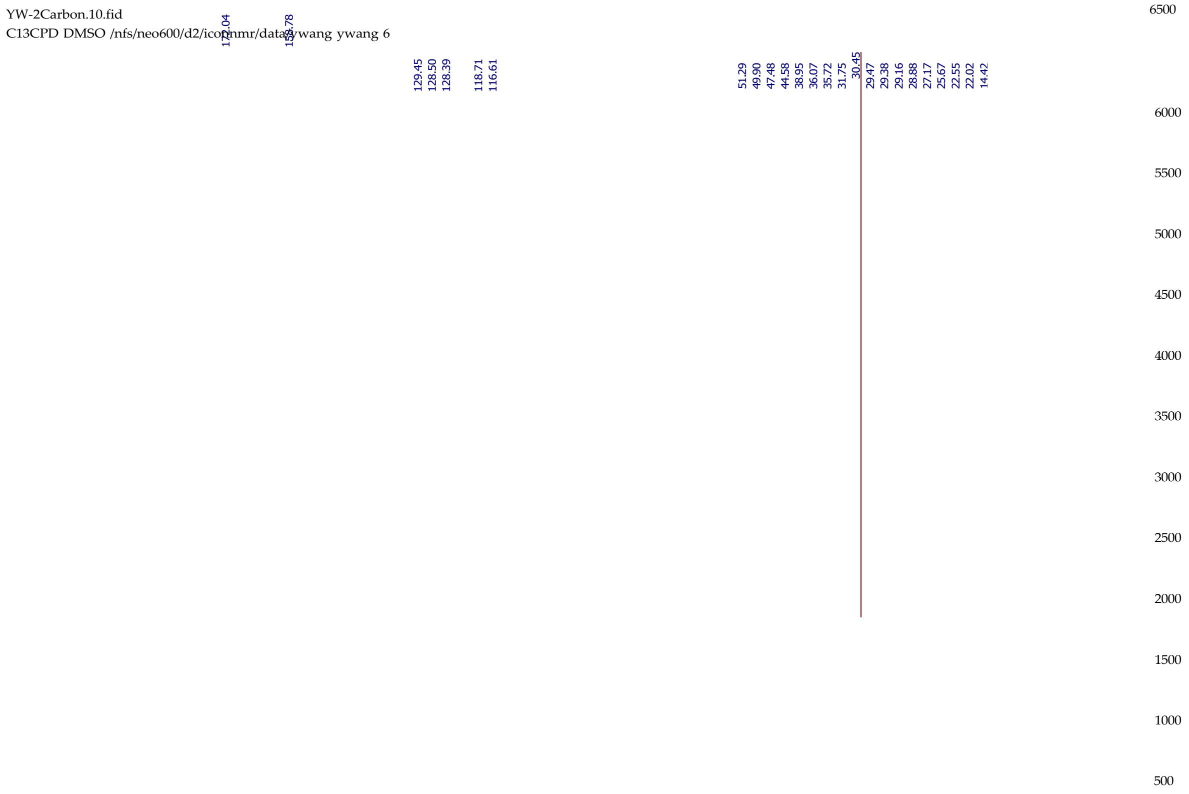
YW-2Carbon.10.fid
C13CPD DMSO /nfs/neo600/d2/iconnmr/data/ywang ywang 6
172.04
158.78
129.45
128.50
128.39
118.71
116.61
51.29
49.90
47.48
44.58
38.95
36.07
35.72
31.75
30.45
29.47
29.38
29.16
28.88
27.17
25.67
22.55
22.02
14.42
6500
6000
5500
5000
4500
4000
3500
3000
2500
2000
1500
1000
500

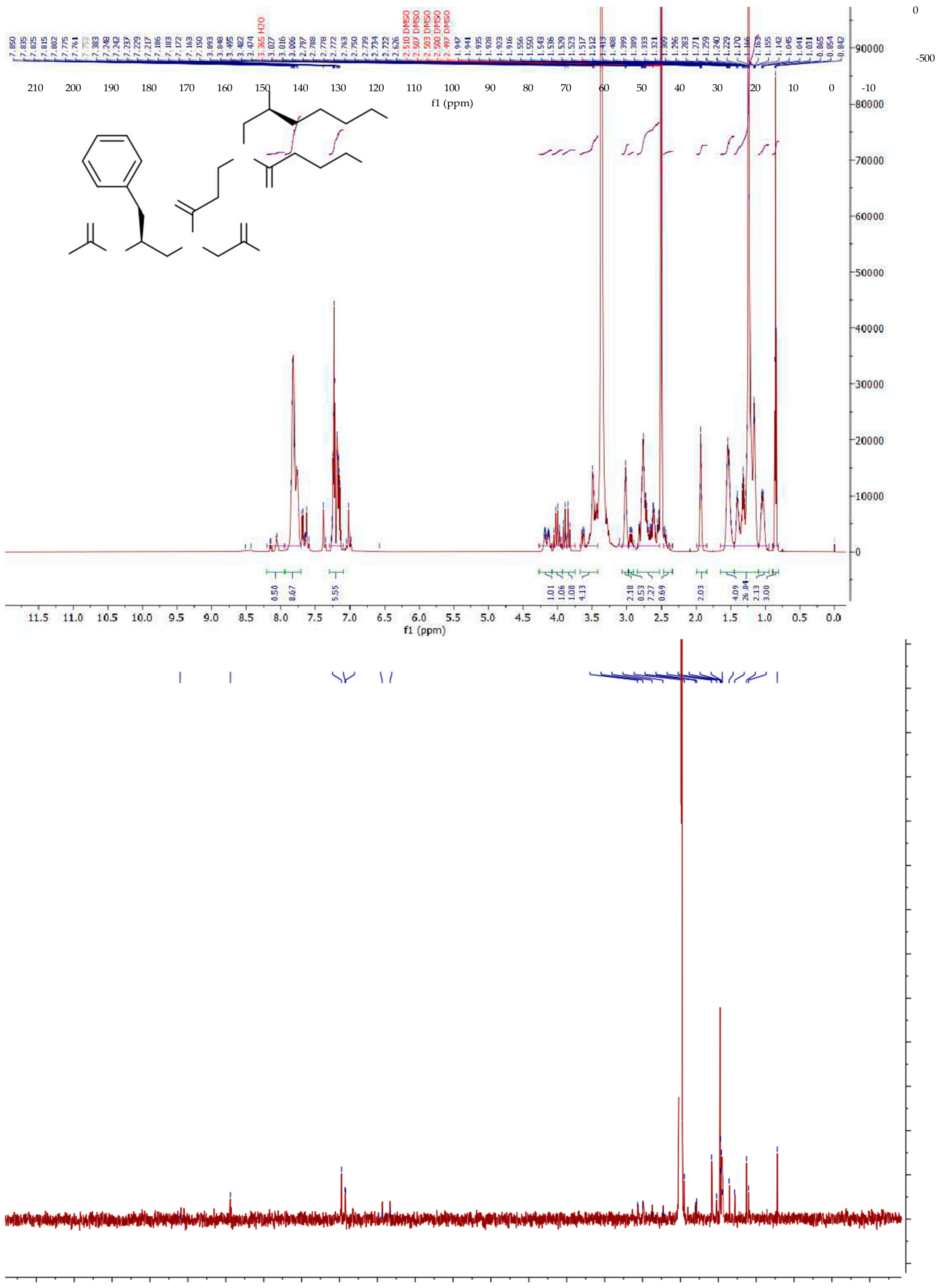

f1 (ppm)
f1 (ppm)

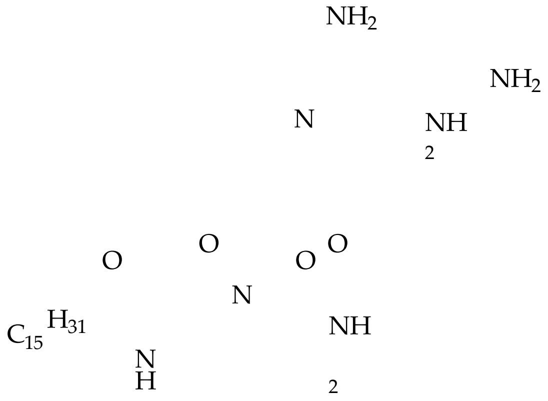

YW-3

YW-3 carbon.10.fid
C13CPD DMSO /mnt/d2/iconnmr/data/sxue sxue 22

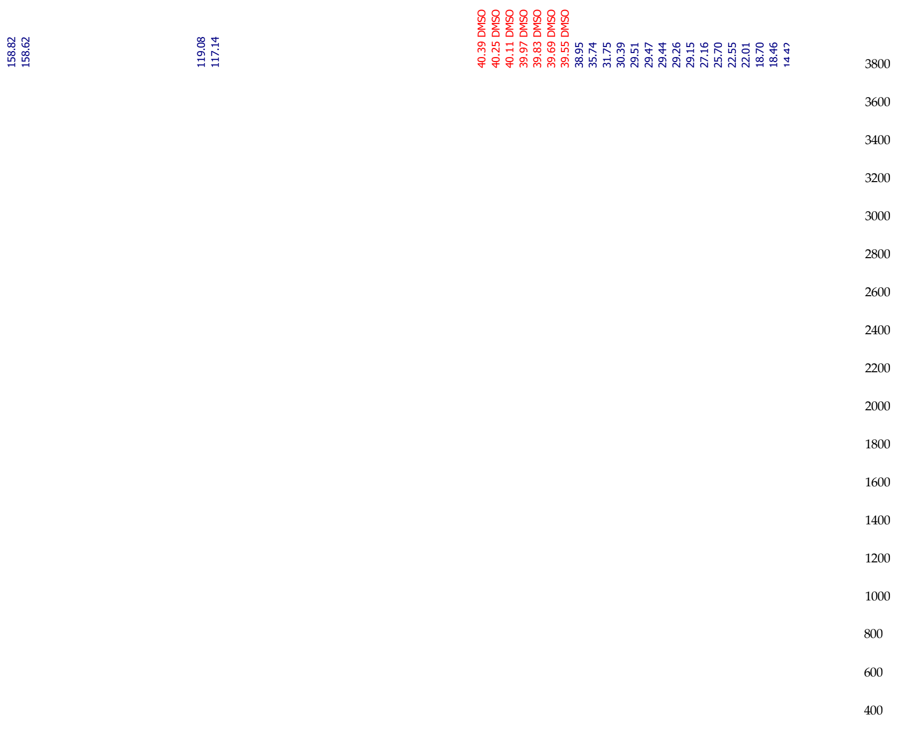

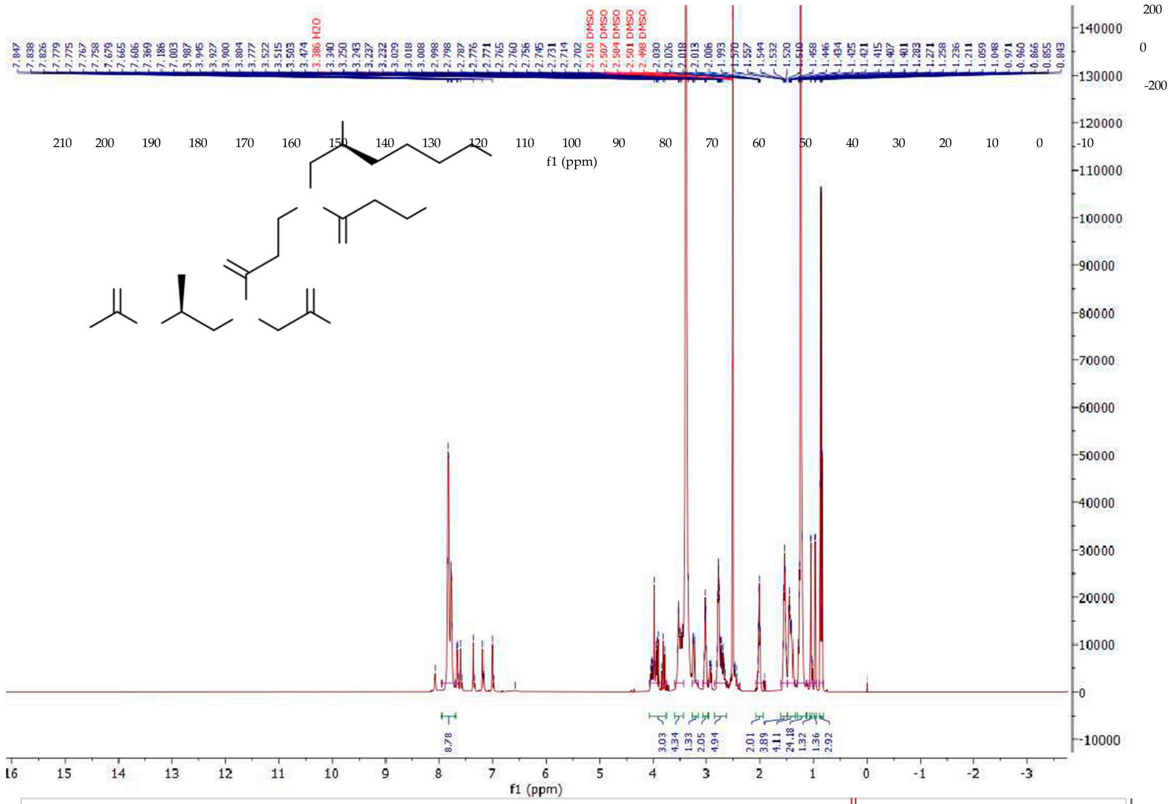

3.386 H2O
2.510 DMSO
2.507 DMSO
2.504 DMSO
2.501 DMSO
2.498 DMSO
210 200 190 180 170 160 150 140 130 120 110 100 90 80 70 60 50 40 30 20 10 0 -10
f1 (ppm)
200
0
-200
140000 130000 120000 110000 100000 90000 80000 70000 60000 50000 40000 30000 20000 10000 0 -10000
8.78
3.03 4.34 1.33 2.05 4.94
2.01 3.89 4.11 24.18 1.32 1.36 2.92
16 15 14 13 12 11 10 9 8 7 6 5 4 3 2 1 0 -1 -2 -3
f1 (ppm)

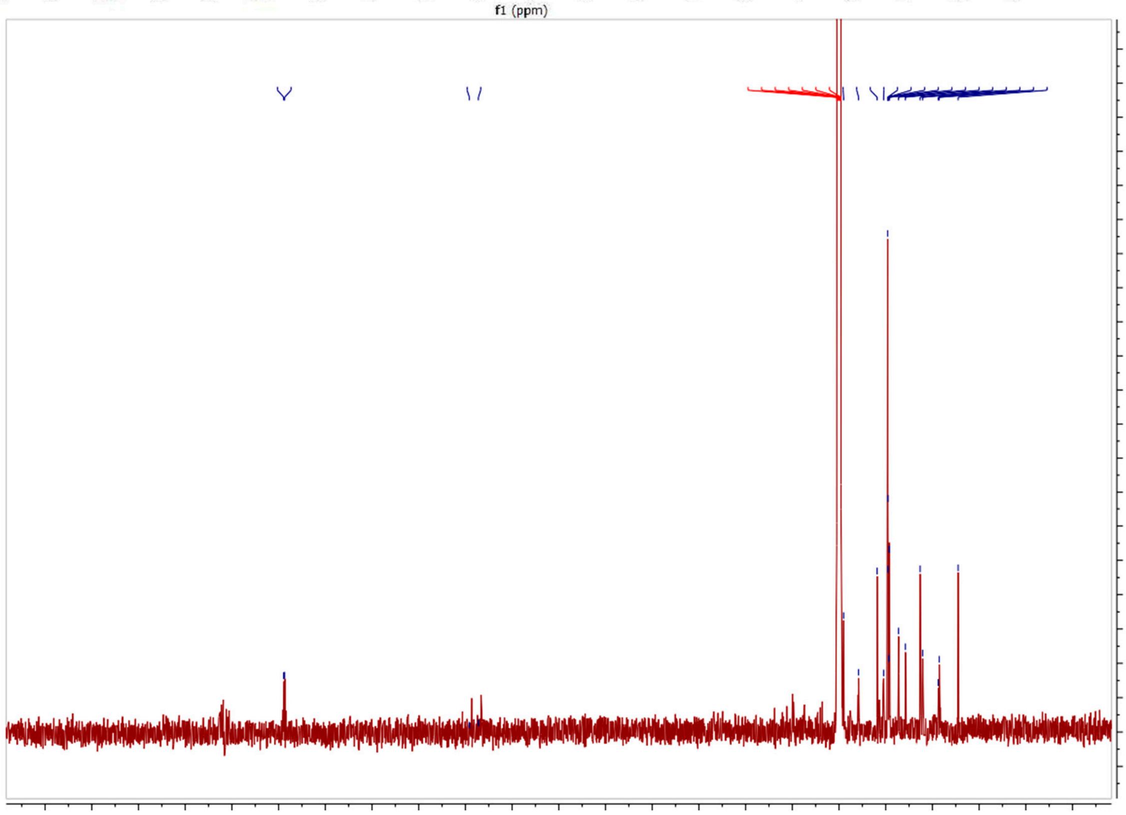

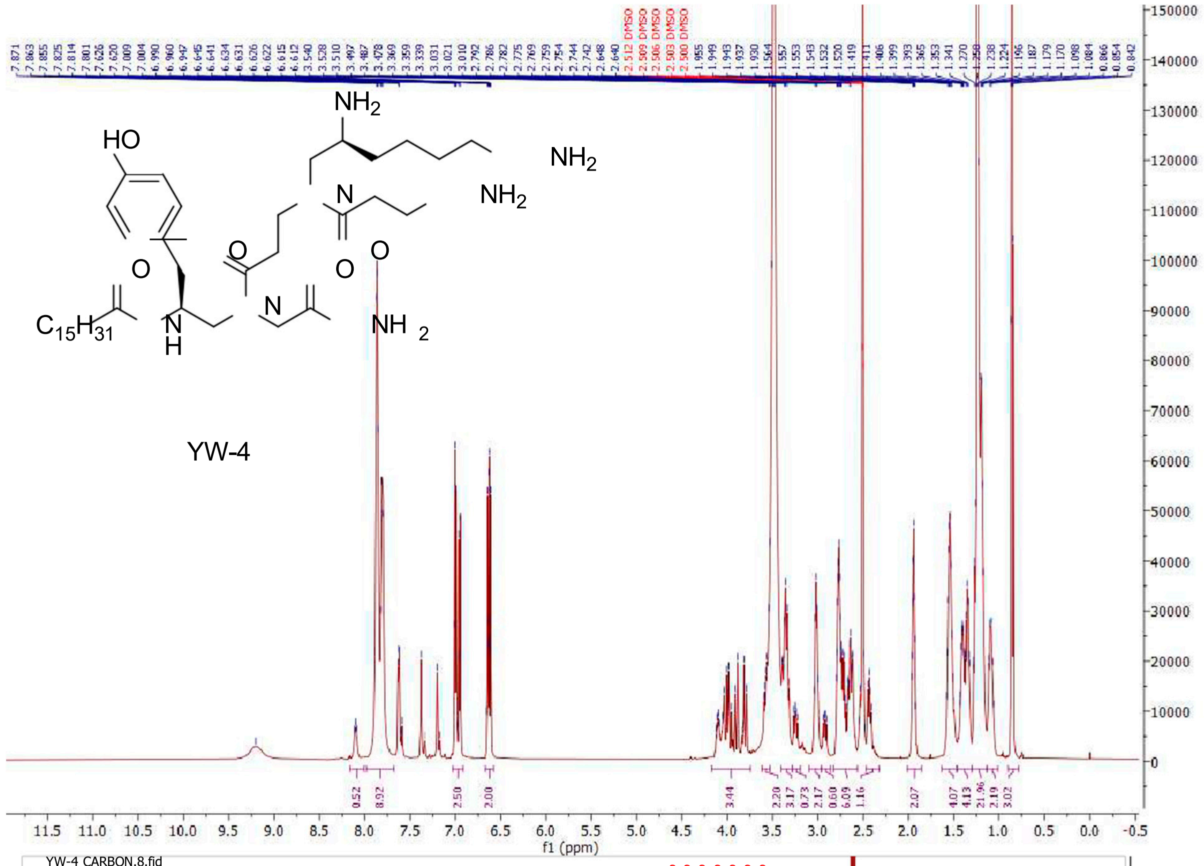
YW-4
HO
NH2
NH2
NH2
NH 2
C15H31
7.871 7.863 7.855 7.825 7.814 7.801 7.626 7.620 7.009 7.004 6.990 6.960 6.947 6.645 6.641 6.634 6.631 6.626 6.622 6.615 6.612 3.540 3.528 3.510 3.497 3.487 3.478 3.369 3.359 3.339 3.031 3.021 3.010 2.792 2.786 2.782 2.775 2.769 2.759 2.754 2.744 2.742 2.648 2.640
2.512 DMSO 2.509 DMSO 2.506 DMSO 2.503 DMSO 2.500 DMSO
1.955 1.949 1.943 1.937 1.930 1.564 1.557 1.553 1.543 1.532 1.520 1.419 1.411 1.406 1.399 1.393 1.365 1.353 1.341 1.270 1.258 1.238 1.224 1.196 1.187 1.179 1.170 1.098 1.084 0.866 0.854 0.842
0.52 8.92 2.50 2.00 3.44 2.20 3.17 0.73 2.17 0.60 6.09 1.16 2.07 4.07 4.13 21.96 2.19 3.02
f1 (ppm)

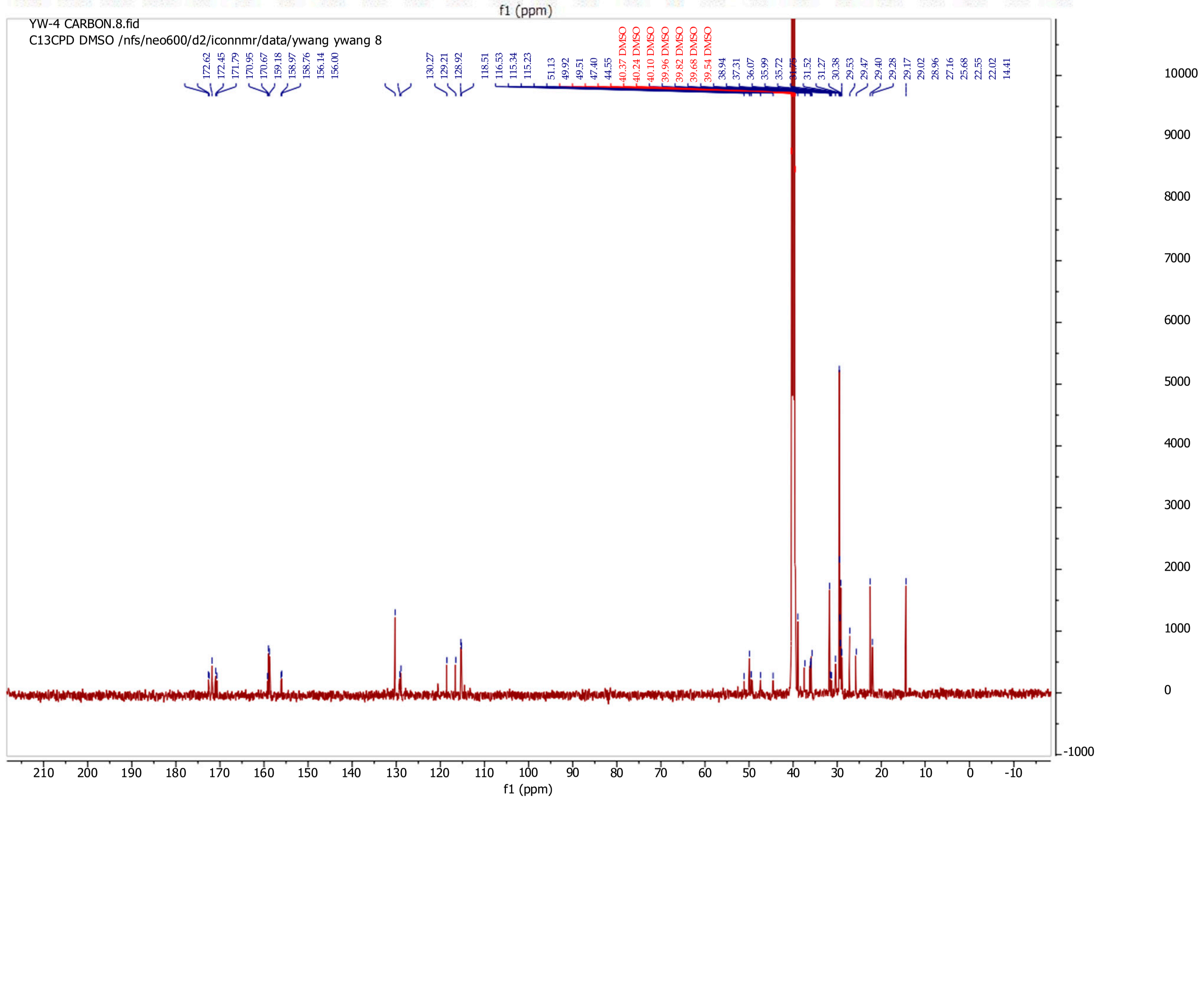
YW-4 CARBON.8.fid
C13CPD DMSO /nfs/neo600/d2/iconnmr/data/ywang ywang 8
172.62 172.45 171.79 170.95 170.67 159.18 158.97 158.76 156.14 156.00 130.27 129.21 128.92 118.51 116.53 115.34 115.23 51.13 49.92 49.51 47.40 44.55
40.37 DMSO 40.24 DMSO 40.10 DMSO 39.96 DMSO 39.82 DMSO 39.68 DMSO 39.54 DMSO
38.94 37.31 36.07 35.99 35.72 31.52 31.27 30.38 29.53 29.47 29.40 29.28 29.17 29.02 28.96 27.16 25.68 22.55 22.02 14.41
f1 (ppm)

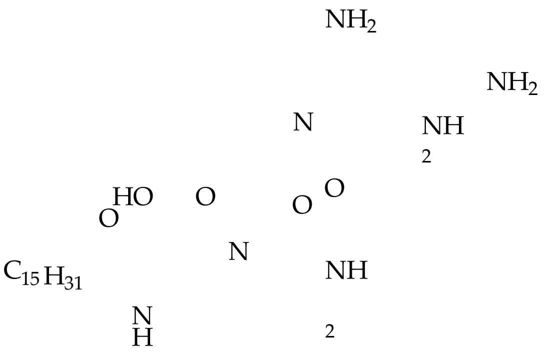

YW-5

YW-5.11.fid
C13CPD DMSO /nfs/neo600/d2/iconnmr/data/ywang ywang 13

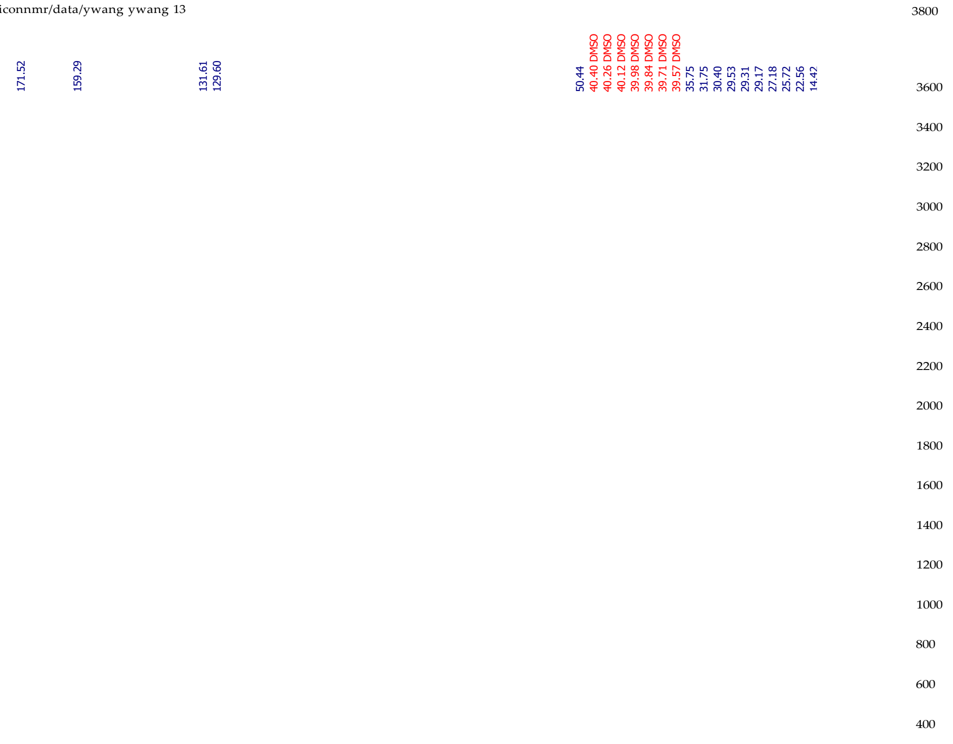

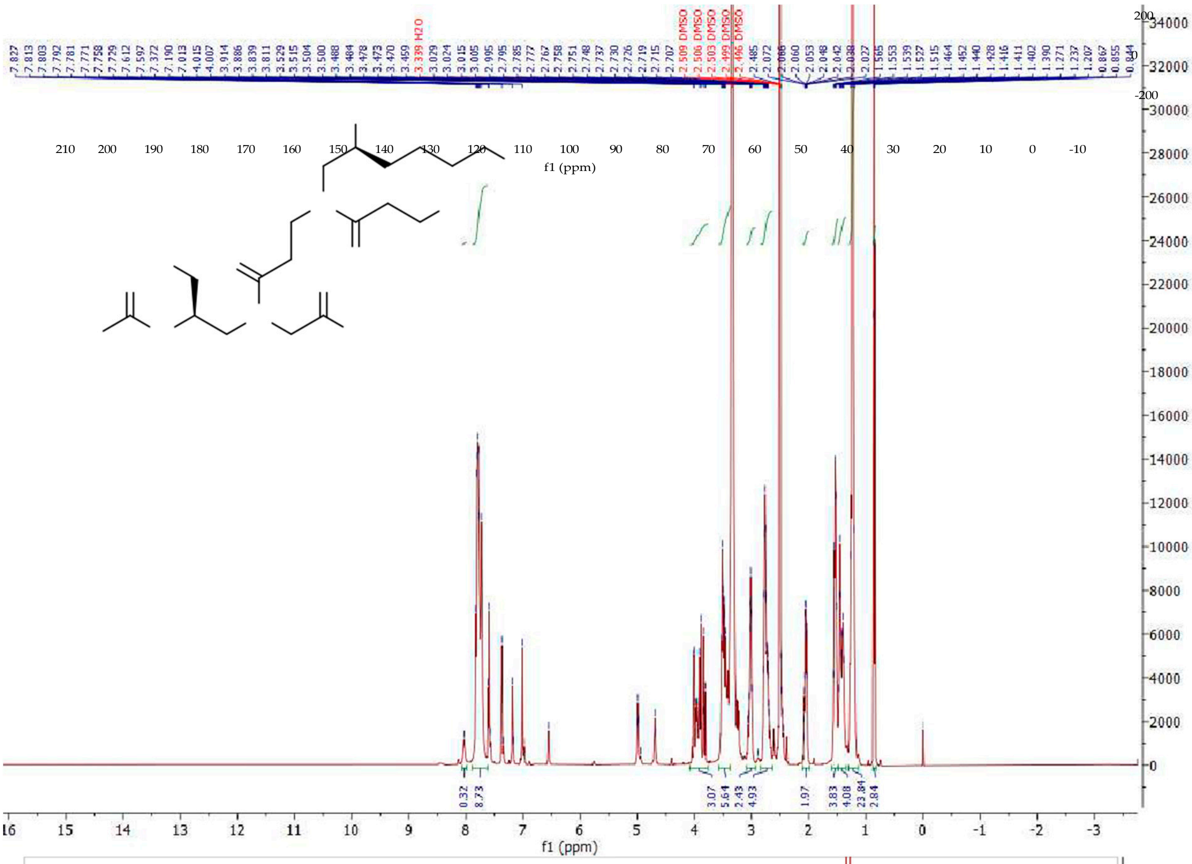
7.827
7.813
7.803
7.792
7.781
7.771
7.758
7.729
7.612
7.597
7.372
7.190
7.013
4.015
4.007
3.914
3.886
3.839
3.811
3.529
3.515
3.504
3.500
3.488
3.484
3.478
3.473
3.470
3.459
3.339 H2O
3.029
3.024
3.015
3.005
2.995
2.795
2.785
2.777
2.767
2.758
2.751
2.748
2.737
2.730
2.726
2.719
2.715
2.707
2.509 DMSO
2.506 DMSO
2.503 DMSO
2.499 DMSO
2.496 DMSO
2.485
2.072
2.066
2.060
2.053
2.048
2.042
2.038
2.027
1.565
1.553
1.539
1.527
1.515
1.464
1.452
1.440
1.428
1.416
1.411
1.402
1.390
1.271
1.237
1.207
0.867
0.855
0.844
0.32
8.73
3.07
5.64
2.43
4.93
1.97
3.83
4.08
23.84
2.84
f1 (ppm)

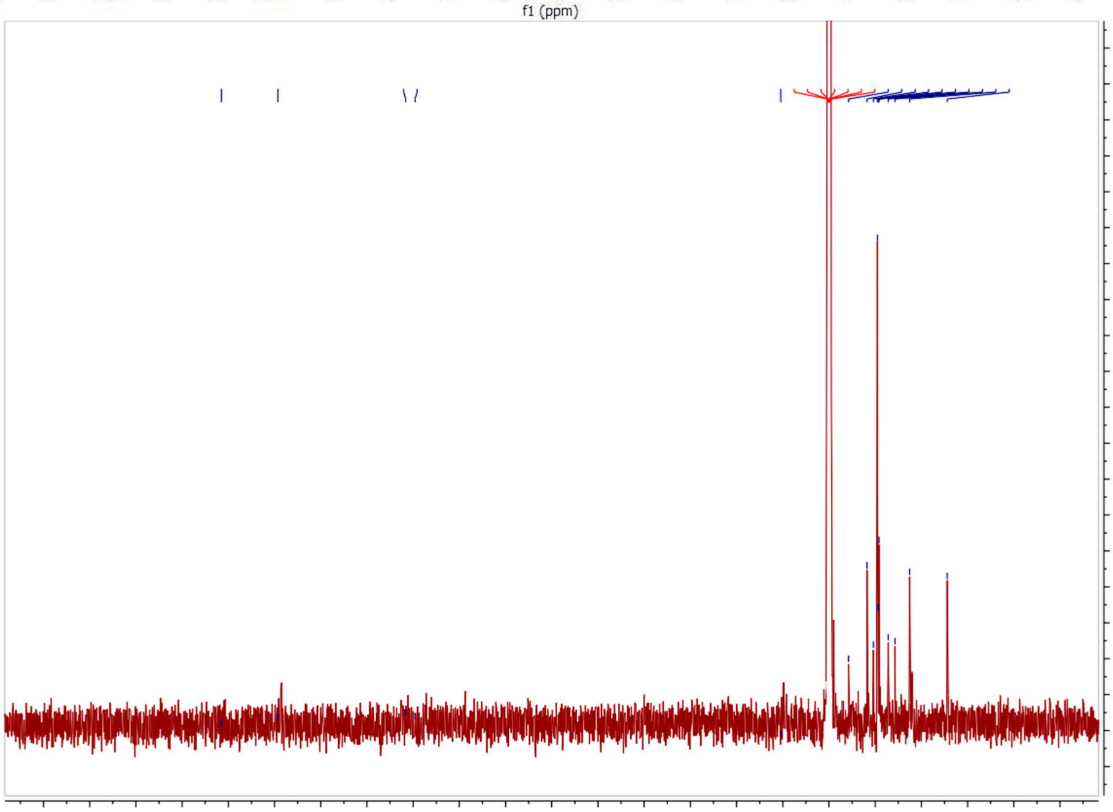

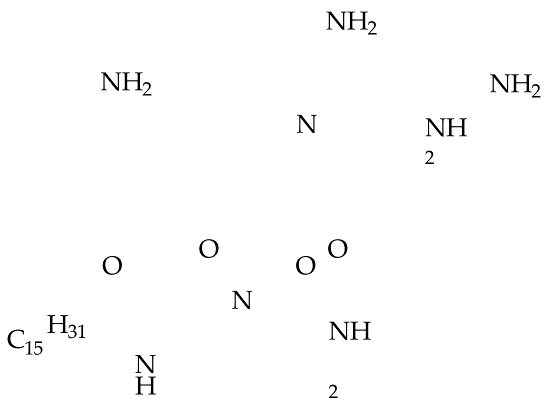

NH2
NH2
NH2
N
NH
2
O
O
O
O
N
C15H31
NH
N
H
2

YW-6

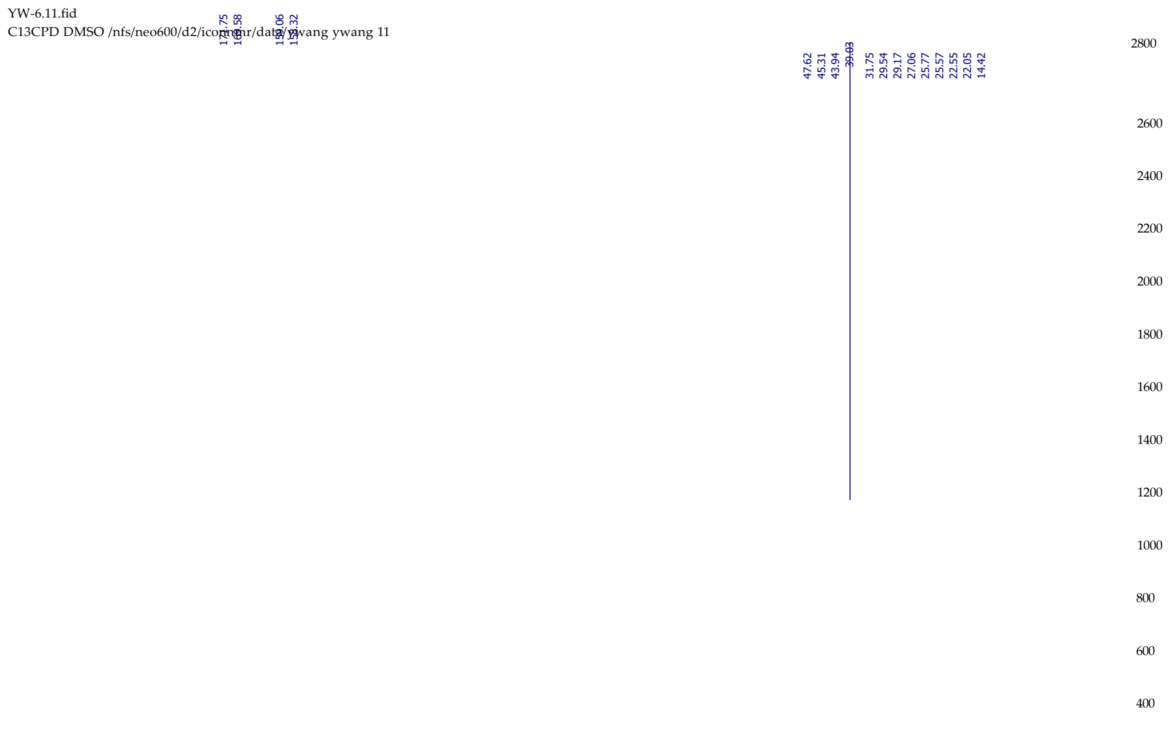

YW-6.11.fid
C13CPD DMSO /nfs/neo600/d2/iconnmr/data/ywang ywang 11
171.75
169.58
159.06
158.32
47.62
45.31
43.94
39.03
31.75
29.54
29.17
27.06
25.77
25.57
22.55
22.05
14.42
2800
2600
2400
2200
2000
1800
1600
1400
1200
1000
800
600
400

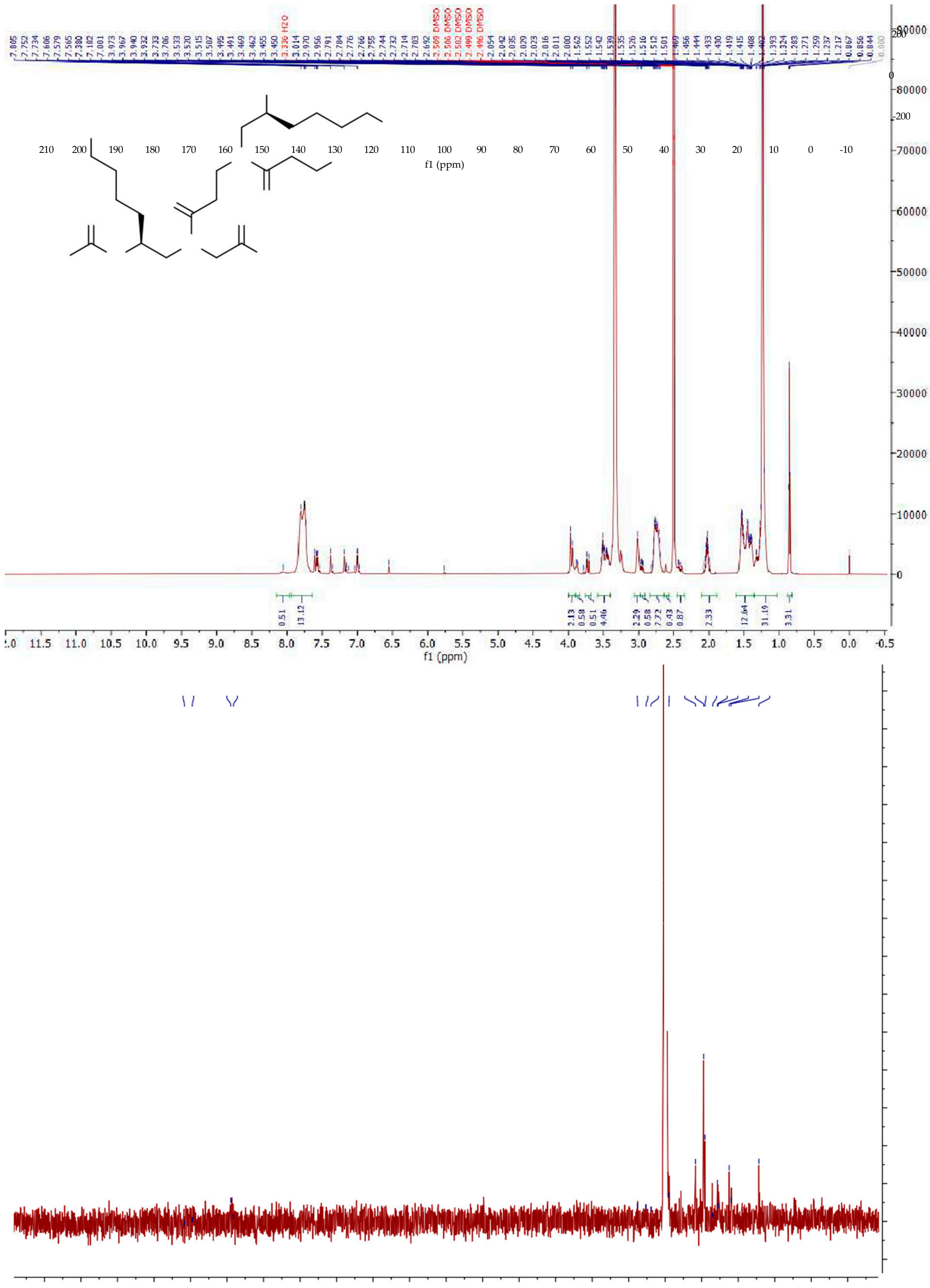

f1 (ppm)
210 200 190 180 170 160 150 140 130 120 110 100 90 80 70 60 50 40 30 20 10 0 -10
f1 (ppm)
11.5 11.0 10.5 10.0 9.5 9.0 8.5 8.0 7.5 7.0 6.5 6.0 5.5 5.0 4.5 4.0 3.5 3.0 2.5 2.0 1.5 1.0 0.5 0.0 -0.5
3.336 H2O
2.509 DMSO
2.506 DMSO
2.502 DMSO
2.499 DMSO
2.496 DMSO
0.51 13.12 2.13 0.58 0.51 4.46 2.29 0.58 7.72 0.43 0.87 2.33 12.64 31.19 3.31

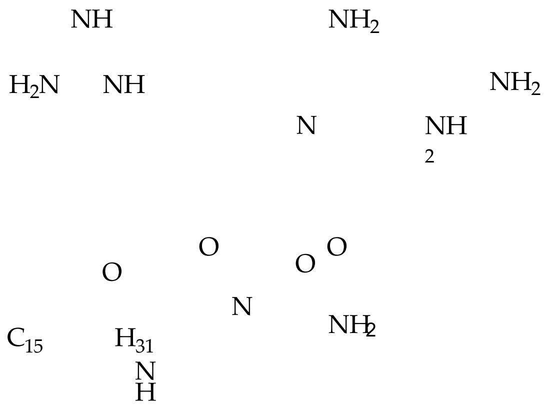
NH
NH2
H2N
NH
NH2
N
NH2
O
O
O
O
N
NH2
C15H31
N
H

YW-7

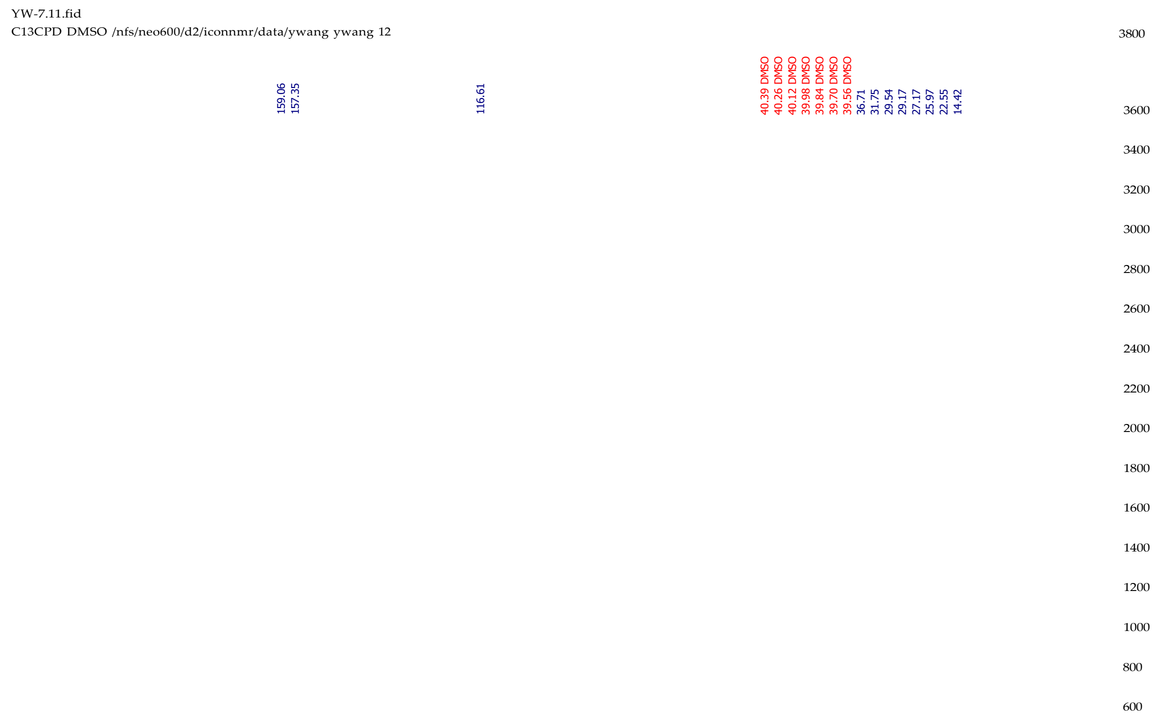
YW-7.11.fid
C13CPD DMSO /nfs/neo600/d2/iconnmr/data/ywang ywang 12
159.06
157.35
116.61
40.39 DMSO
40.26 DMSO
40.12 DMSO
39.98 DMSO
39.84 DMSO
39.70 DMSO
39.56 DMSO
36.71
31.75
29.54
29.17
27.17
25.97
22.55
14.42
3800
3600
3400
3200
3000
2800
2600
2400
2200
2000
1800
1600
1400
1200
1000
800
600

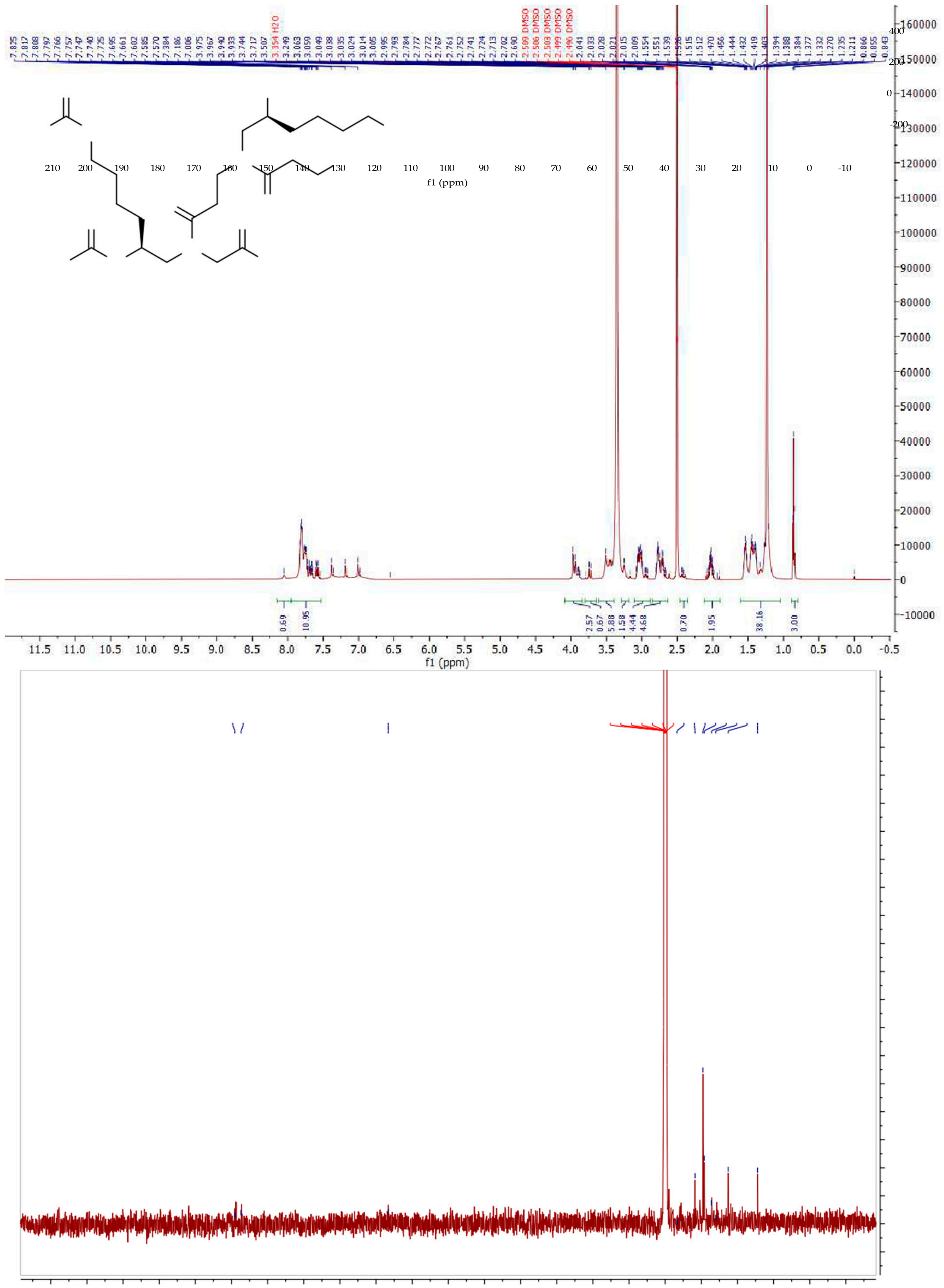
7.825 7.817 7.808 7.797 7.766 7.757 7.747 7.740 7.725 7.695 7.661 7.602 7.585 7.570 7.384 7.186 7.006 3.975 3.967 3.940 3.933 3.744 3.717 3.507
3.354 H2O
3.249 3.063 3.059 3.049 3.038 3.035 3.024 3.014 3.005 2.995 2.793 2.784 2.777 2.772 2.767 2.761 2.752 2.741 2.724 2.713 2.702 2.690
2.509 DMSO 2.506 DMSO 2.503 DMSO 2.499 DMSO 2.496 DMSO
2.041 2.033 2.028 2.021 2.015 2.009 1.554 1.551 1.539 1.526 1.515 1.512 1.470 1.456 1.444 1.432 1.419 1.403 1.394 1.388 1.384 1.377 1.332 1.270 1.235 1.211 0.866 0.855 0.843
0.69 10.95 2.57 0.67 5.88 1.58 4.44 4.68 0.70 1.95 38.16 3.00
f1 (ppm)

3. *Table S1: HRMS data*

**Peptide**	**HRMS (ESI) [Exact Mass+H$^+$] calculated**	**HRMS (ESI) [Exact Mass+H$^+$] found**
YW-1	667.5742	668.5747
YW-2	701.5568	702.5604
YW-3	625.5255	626.5286
YW-4	717.5517	718.5532
YW-5	641.5204	642.5214
YW-6	682.5833	683.5852
YW-7	724.6051	725.6050
YW-8	673.5255	674.5298
YW-9	645.4942	646.4979
YW-10	617.4629	618.4668
YW-11	589.4316	590.4355

References

1. Singh, S.; Wang, M.; Gao, R.; Teng, P.; Odom, T.; Zhang, E.; Xu, H.; Cai, J.; Hu, Y.; Amin, M.N.; et al. Lipo-γ-AApeptides as a New Class of Potent and Broad-Spectrum Antimicrobial Agents. *J. Med. Chem.* **2012**, *55*, 4003–4009. https://doi.org/10.1021/jm300274p
